# The effect of ursodeoxycholic acid on the relative expression of the lipid metabolism genes in mouse cholesterol gallstone models

**DOI:** 10.1186/s12944-020-01334-3

**Published:** 2020-07-02

**Authors:** Ning Fan, Ke Meng, Yuqing Zhang, Yong Hu, Donghua Li, Qiaoying Gao, Jianhua Wang, Yanning Li, Shangwei Wu, Yunfeng Cui

**Affiliations:** 1 Beichen Chinese Medicine Hospital Affiliated to Tianjin University of Traditional Chinese Medicine, 436 Jingjin Road, Beichen District, Tianjin, 300400 China; 2 Department of Obstetrics and Gynecology, General Hospital of Tianjin Medical University, 154 AnShan Road, HePing District, Tianjin, 300052 China; 3 Department of Surgery, Tianjin Nankai Hospital, Nankai Clinical School of Medicine, Tianjin Medical University, 122 Sanwei Road Nankai District, Tianjin, 300100 China; 4 Tianjin Medical University, 22 Qixiangtai Road, Heping District, Tianjin, 300070 China; 5 Institute of Acute Abdomen in Integrative Medicine, Tianjin Nankai Hospital, Nankai Clinical School of Medicine, Tianjin Medical University, 122 Sanwei Road Nankai District, Tianjin, 300100 China

**Keywords:** Cholesterol gallstone, Lipid metabolism, Ursodeoxycholic acid, ABCG8, CYP7A1, CYP27A1, LXR, PPAR-α, ABCB11

## Abstract

**Background:**

Many studies indicate that gallstone formation has genetic components. The abnormal expression of lipid-related genes could be the basis for particular forms of cholesterol gallstone disease. The aim of this study was to obtain insight into lipid metabolism disorder during cholesterol gallstone formation and to evaluate the effect of ursodeoxycholic acid (UDCA) on the improvement of bile lithogenicity and its potential influence on the transcription of lipid-related genes.

**Methods:**

Gallstone-susceptible mouse models were induced by feeding with a lithogenic diet (LD) for 8 weeks. Bile and liver tissues were obtained from these mouse models after 0, 4 and 8 weeks. Bile lipids were measured enzymatically, and the cholesterol saturation index (CSI) was calculated to evaluate the bile lithogenicity by using Carey’s critical tables. Real-time polymerase chain reaction (RT-PCR) was used to detect the mRNA expression levels of farnesoid X receptor (FXR), liver X receptor (LXR), adenosine triphosphate-binding cassette subfamily G member 5/8 (ABCG5/8), cholesterol 7-α hydroxylase (CYP7A1), oxysterol 7-α hydroxylase (CYP7B1), sterol 27-α hydroxylase (CYP27A1), peroxisome proliferator-activated receptor alpha (PPAR-α) and adenosine triphosphate-binding cassette subfamily B member 11 (ABCB11).

**Results:**

The rate of gallstone formation was 100% in the 4-week group but only 30% in the UDCA-treated group. The UDCA-treated group had a significantly lower CSI compared with other groups. Of special note, the data on the effects of UDCA showed higher expression levels of ABCG8, ABCB11 and CYP27A1, as well as lower expression levels of LXR and PPAR-α, compared to the model control group.

**Conclusions:**

UDCA exhibits tremendously potent activity in restraining lipid accumulation, thus reversing the lithogenic effect and protecting hepatocytes from serious pathological damage. The abnormal expression of ABCG8, CYP7A1, CYP27A1, LXR and PPAR-α might lead to high lithogenicity of bile. These results are helpful in exploring new lipid metabolism pathways and potential targets for the treatment of cholesterol stones and for providing some basis for the study of the pathogenesis and genetic characteristics of cholelithiasis. Research on the mechanism of UDCA in improving lipid metabolism and bile lithogenicity may be helpful for clinical treatment and for reducing the incidence of gallstones.

## Background

It is believed that the prevalence of gallstone disease (GSD) ranges from 10 to 30%, which has been observed in multiple studies [1, 2]. Due to the improvement in living standards and irrational eating habits [3], GSD has become one of the most widespread gastroenterological conditions, imposing a considerably socioeconomic burden on health care [4]. In addition, GSD is correlated with Mirizzi syndrome and insulin resistance [5, 6] and can extend into the liver, gallbladder and gastrointestinal tract. More than 90% of gallstones consist mainly of cholesterol and are formed within the gallbladder [7]. It is generally thought that the key pathological abnormalities are associated with an imbalance of gallbladder bile with cholesterol supersaturation relative to bile salts. In addition, phospholipids and gallbladder hypomotility are considered as prerequisites to gallstone disease [8, 9].

In 1882, Dr. Langenbuch performed the first cholecystectomy, which has been considered a significant routine surgery to eradicate gallstones. With the development of surgical technology, a laparoscopic cholecystectomy has become one of the most commonly performed surgical procedures worldwide [10]. However, cholecystectomy is invasive and has a risk of postoperative complications, and not all patients with symptomatic gallstones are candidates for surgery [11]. UDCA, a natural dihydroxy bile acid, promotes gallstone dissolution and has been attributed to have several other beneficial effects, which significantly affect the treatment of cholelithiasis [12]. Currently, UDCA is recommended as a pharmacological therapy to prevent gallstone formation primarily since it is noninvasive and has fewer complications [13]. In addition, increasing studies support the notion that UDCA may enhance gallbladder muscle contractility and gastrointestinal motility to empty the gallbladder, stomach and intestine [14, 15]. In recent decades, there have been numerous investigations emphasizing the association of lipid-related genes with GSD, although little is known about how UDCA influences nuclear receptors during the process of GSD [16].

Nuclear receptors include FXR, LXR and PPAR-α, which are hepatic lipid regulatory transcription factors of the candidate genes for gallstones [17, 18]. It is plausible that nuclear receptors are associated with the molecular mechanism of gallstone dissolution during UDCA intervention [7]. CYP7A1 is a rate-limiting enzyme of the classic pathway of bile acid biosynthesis and plays a significant role in the alternative pathway to catalyze cholesterol into chenodeoxycholic acid (CDCA) or cholic acid (CA). FXR inhibits bile acid synthesis through downregulating CYP7A1, whereas LXR may upregulate the transcription of CYP7A1 mRNA. On the other hand, LXR regulates ABCG5 and ABCG8 to facilitate cholesterol efflux [19–21]. Moreover, adenosine triphosphate-binding cassette subfamily B member 4 (ABCB4) and ABCB11 are determinants of phospholipids (PL) and total bile acid (TBA) secretion, both of which act as flippases and efflux transporters to mediate movement of PL and TBA from hepatocytes to the canalicular membrane. TBA is reabsorbed at the terminal ileum into the portal vein to complete the enterohepatic circulation of bile acid.

This study aimed to investigate the effect of an LD and an abnormal lipid metabolism on the development of gallstones in the liver and gallbladder, histological changes in the liver, alteration in bile composition and the relative mRNA expression of genes relating to the regulation of lipid and bile acid metabolism in a mouse model of gallstone disease over a period of 8 weeks. In addition, UDCA was used in this LD-induced gallstone disease mouse model and compared with control models to evaluate a potential mechanism of action.

## Methods

### Animals and diets

This study was approved by the Animal Care and Use Committee in Tianjin Nankai Hospital (TMUh-MEC2012019). Healthy male C57BL/6 J mice (8 weeks old, 18–20 g) were purchased from the Beijing HFK Biotechnology Company Limited (Beijing, China) and housed in plastic cages with environmentally controlled conditions (22 ± 2 °C, a 12 h light cycle) with water and food ad libitum. After adaptive feeding for 2 weeks, 60 mice were divided randomly into 6 groups, with 10 mice in each group: LD 0-week group, LD 4-week group, LD 8-week group, LD+ normal saline (NS) 8-week group, regular rodent diet (RD) + NS 8-week group and LD + UDCA 8-week group (Table 1). The first 3 groups received an LD for 0, 4, and 8 weeks, respectively, and the last 3 groups were given the same volume of NS or UDCA (60 mg/kg/d, once a day) at 9:00 am for 8 consecutive weeks. The “0 + LD” group and “RD + NS” group represent the control groups. The “8 + LD” group and “LD + NS” group represent the model groups.

**Table 1:** Mouse grouping and feeding schedule

Groups	Number	Feeding time
0 + LD	10	0 week
4 + LD	10	4 weeks
8 + LD	10	8 weeks
RD + NS	10	8 weeks
LD + NS	10	8 weeks
LD + UDCA	10	8 weeks

The LD consisted of 80.25% regular rodent diet, 0.50% cholic acid (VETEC, Kenilworth, NJ, USA), 1.25% cholesterol (Beijing Solarbio Science & Technology, China), 16% butterfat and 2% corn oil. All procedures conformed to the Animal Care and Use Committee of Tianjin Nankai Hospital.

### Bile, gallstone, and liver sample collections

At the end of each experiment, following an overnight fasting, the animals were anesthetized with 4% chloral hydrate. A mid-abdomen incision was made, after which liver tissues, gallstone, and bile were collected. Portions of the liver tissues were obtained for histopathological analysis and RT-PCR detection. Bile specimen was aspirated and kept for the subsequent analysis of lipid profiles. The remaining liver tissues and gallstones were stored at − 80 °C (Thermo Scientific Forma, Waltham, MA, USA) for further assessment. After the collection of samples, the mice were euthanized.

### Hematoxylin and eosin (HE) staining

Liver tissues were fixed with 4% neutral buffered formaldehyde for 2 weeks. The fixed tissues were dehydrated, embedded in paraffin, and serially sectioned into 4 μm slices to eventually achieve HE staining for subsequent observation under an upright microscope (Nikon, Chiyoda ward, Kyoto, Japan).

### Analysis of bile lipids

Bile total cholesterol (TC), PL and TBA (the TC diagnostic kit was purchased from Roche Diagnostics Ltd., Basel, Switzerland; the PL kit was purchased from Beijing Leadman Biochemical Co, Ltd., China; the TBA kit was purchased from Beijing Strong Biotechnologies, Inc., China) were detected by an automatic biochemical analyzer (Cobas 8000, Basel, Switzerland). The CSI was calculated according to Carey’s critical tables [22].

### RT-PCR detection

Total RNA was extracted by using an RNAprep Pure Tissue Kit (Tiangen, Beijing, China). To harvest cDNA, 2 μg RNA and the Oligo (dT) 18 primer was used in the reverse transcription reaction using the RevertAid™ First Strand cDNA Synthesis Kit (Thermo, USA). Amplification was performed on a RT-PCR detection system (Bio-Rad IQ5, Waltham, MA, USA) with the GoTaq® qPCR Master Mix (Promega, Madison, Wisconsin, USA). The relative expression levels of genes were quantified by using the 2^–ΔΔCt^ method, and glyceraldehyde-3-phosphate dehydrogenase (GAPDH) was deemed as the invariant control. Ct represents cycle threshold. The forward and reverse primers of the mouse genes were designed for RT-PCR and gel electrophoresis as follows:

FXR (5′-TGGGTACCAGGGAGAGACTG-3′ and 5′-GTGAGCGCGTTGTAGTGGTA-3′),

LXR (5′-AGACGTCACGGAGGTACAAC-3′ and 5′-AGCAGAGCAAACTCAGCATC-3′),

ABCG5 (5′-GGAGAACATTGAAAGAGCAC-3′ and 5′-GTTACTCGCCTCAGCAG-3′),

ABCG8 (5′-GACAGCTTCACAGCCCACAA-3′ and 5′-GCCTGAAGATGTCAGAGCGA-3′),

CYP7A1 (5′-CTTCATCACAAACTCCCTGTC-3′ and 5′-GTCCAAATGCCTTCGCAG-3′),

CYP7B1 (5′-CCGATTCTGCCGTCTCCTT-3′ and 5′-CCAGCCTTACTCTGCAAAGCTT-3′),

CYP27A1 (5′-GATCTTCATCGCACAAGGAG-3′ and 5′-GATAACCTCGTTTAAGGCATCC-3′),

PPAR-α (5′-TGGTTGAATCGTGAGGAACA-3′ and 5′-ATCGCCACTAAGGTGTCAGG-3′),

ABCB11 (5′-AATAGACAGGCAACCCGT-3′ and 5′-GAGAAGGATAATGGAAGGTCAC-3′).

In addition, the relative expression levels of the genes’ mRNAs were normalized to GAPDH mRNA.

### Statistical analysis

The data were presented as the mean ± standard deviation. A chi-square test was used to compare the stone formation rate among the control group, model group and LD + UDCA group. Comparisons among the different groups were determined by one-way analysis of variance with least significant difference (LSD) and Student-Newman-Keuls (SNK) analysis. The enumeration data were expressed as a percentage. Statistical analysis was delineated on SPSS 17.0 (SPSS, Chicago, IL, USA) and GraphPad Prism 6.0 (GraphPad Software, La Jolla, CA, USA). A difference was considered statistically significant when the two-sided tests showed a *P* value of less than 0.05 and extremely significant when *P* < 0.01 or *P* < 0.001. A Spearman correlation coefficient analysis was performed to correlate bile lipid concentrations with gene expression levels.

## Results

### The evolution of gallstone formation

**Fig. 1:**
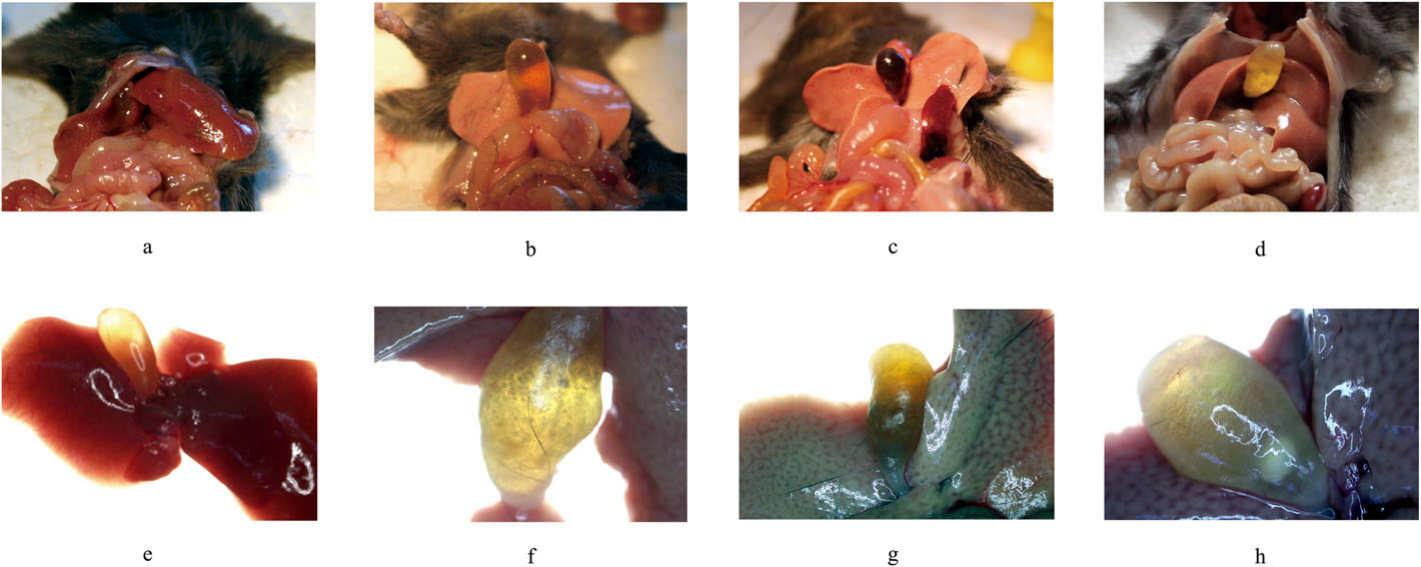
The evolution of gallstone formation in the liver and gallbladder. **a**, **b**, **c**, and **d**: the macroscopic anatomic structure of the liver and gallbladder; **e**, **f**, **g**, and **h**: images from the 2× stereo microscope; **a** and **e**: normal liver and gallbladder; **b**: cholecystectasia; **f**: floccules; **c** and **g**: cholestasis; **d** and **h**: massive gallstone

The conditions of the liver and gallbladder with or without gallstones were recorded completely in macroscopic and microscopic evaluations (2× stereo, Olympus, Kyoto, Japan) and are pictured in Fig. 1.

The conditions in the samples of the 0 + LD and RD + NS groups appeared to be normal, which showed dark red liver tissues and transparent gallbladders with no flowing gallstones, floccules or crystals. In the 4 + LD group, mild changes appeared in the liver, with a shallow color, crisp texture and serrated edge, while the gallbladders were significantly altered with cholecystectasia and cholestasis, accompanied by flocculent sediment. In the model groups, massive granulated white and light-yellow gallstones floated in bile, and dramatic changes occurred in the liver, which turned gray or white with a greasy appearance. Otherwise, UDCA reversed the evolution of gallstone formation, improving the conditions of cholecystectasia and cholestasis, which were similar to the 4 + LD group.

### Gallstone prevalence

**Fig. 2:**
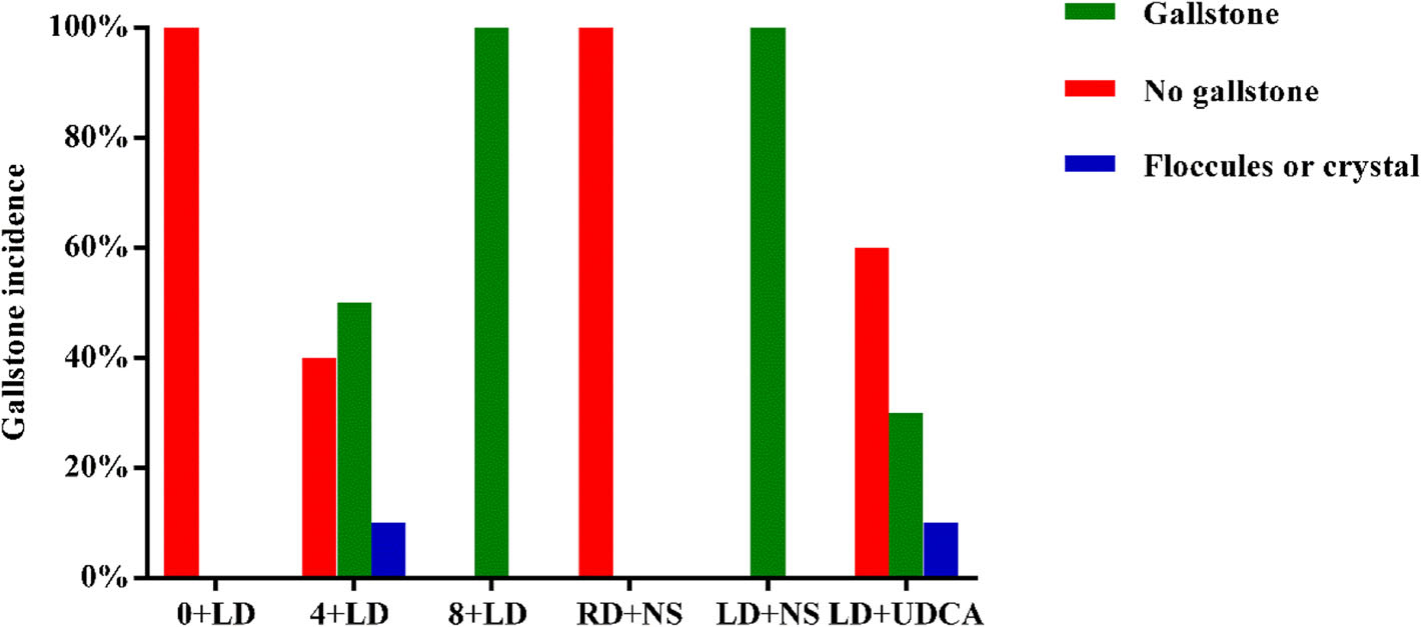
Gallstone prevalence in each group (*n* = 10)

The preliminary experiment was performed on 10 C57BL/6 J mice on an LD for 8 weeks to obtain a successful mouse model of cholesterol gallstones. As shown in Fig. 2, the gallstone prevalence in the 4 + LD group, control group and model group were 50, 0 and 100% (5/10, 0/10, and 10/10), respectively. The prevalence rate of the LD + UDCA group was 30% (3/10), which was significantly lower than that of the LD + NS group. Floccules were viewed as the transition state of gallstone formation, and the prevalence in both the 4 + LD group and LD + UDCA group was 10% (1/10). Statistical analysis showed that the prevalence of gallstones was significantly different among the model group, control group and LD + UDCA group (*P*<0.05).

### Qualitative analysis of gallstones

Gallstones were collected and analyzed by Fourier transform infrared spectroscopy (Thermo Scientific Forma, Waltham, MA, USA). The collected gallstones were washed and dried to perform qualitative analysis with Fourier transform infrared spectroscopy, and a strong absorption peak of the cholesterol gallstone was detected. There were strong absorption peaks detected at 2930, 1466, 1382 and 1055 cm^− 1^, which aligned with the cholesterol absorption peaks. There was no characterized pigment absorption peak, indicating that the gallstones can be classified as cholesterol gallstones.

### Morphological observation

**Fig. 3:**
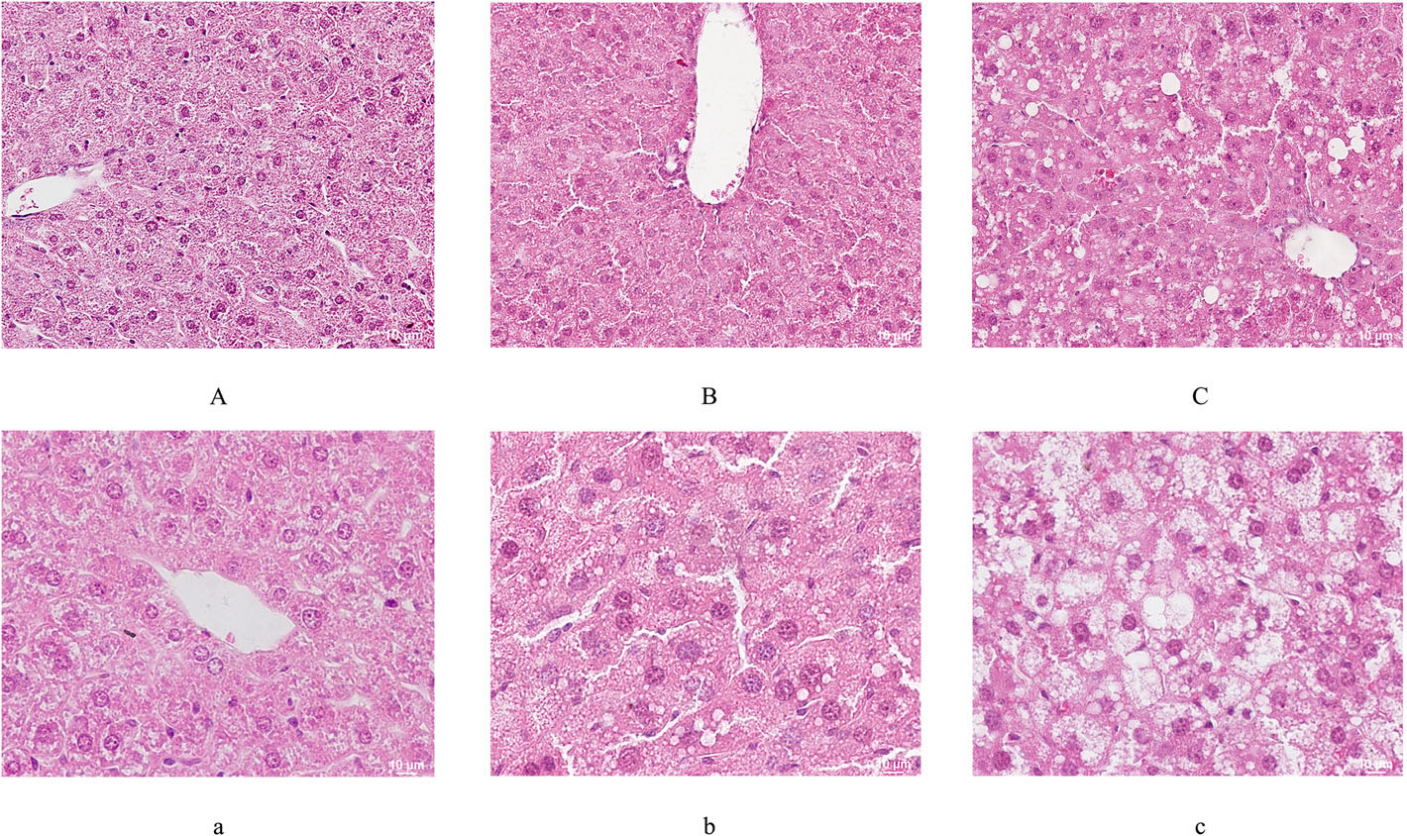
HE staining of liver tissues. **A** and **a**: normal liver cell; **B** and **b**: cell in transition stage; **C** and **c**: cell with serious damage. The magnifications are 200× in **A**, **B** and **C** and 400× in **a**, **b** and **c**

As a result of the LD, the histopathology of the liver tissue was worse than that observed in the control groups (Fig. 3). Fatty degeneration of the liver cells, ballooning degeneration of the liver cells or hepatocellular hyperplasia could be markedly observed in the model groups. Regarding reduction in the fatty droplets in the hepatocytes, the morphological change in the LD + UDCA group and the 4 + LD group fell between that of the control group and the model group.

### Bile lipid levels

**Fig. 4:**
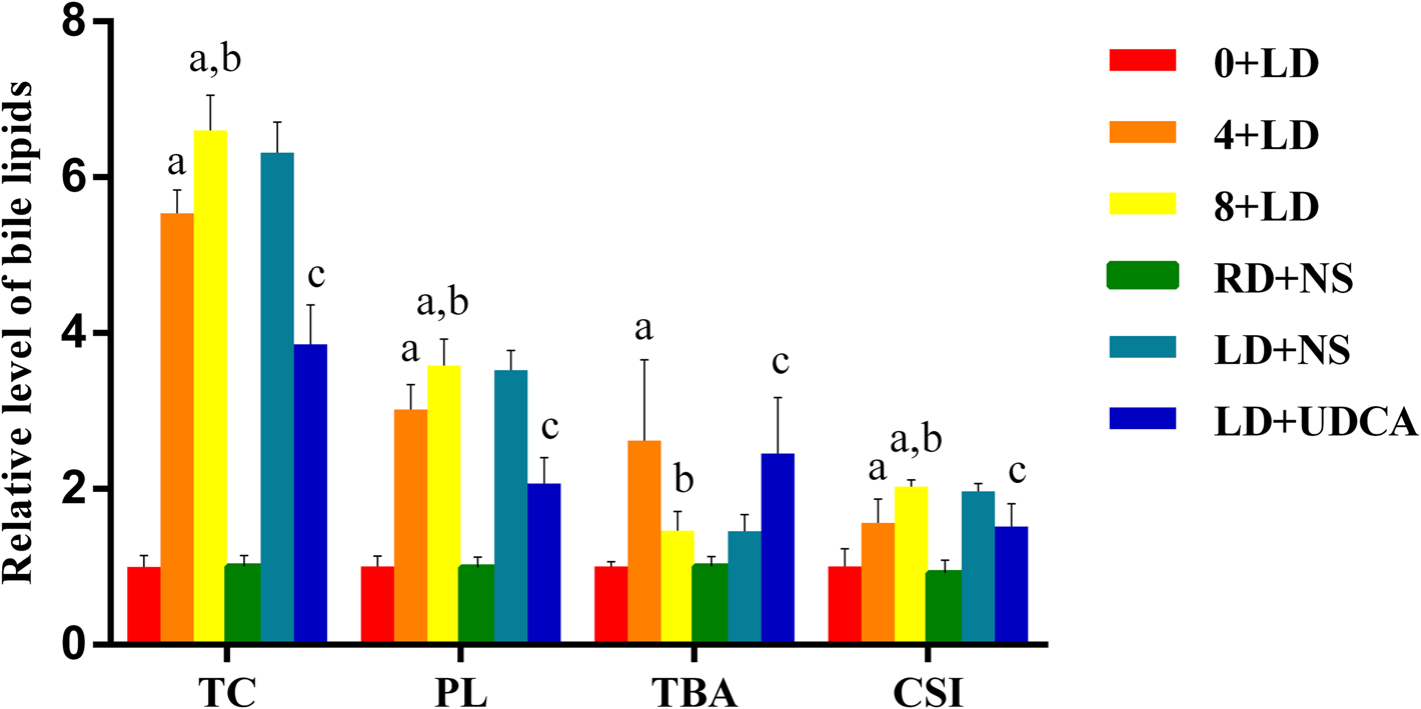
Relative levels of the bile lipids. a, significantly different from the control group (*P* < 0.001); b, significantly different from the 4 + LD group (*P* < 0.001); c, the *P* value in LD + UDCA group was significantly different from that of the control group (*P* < 0.001). The data of the lipid contents of the control group were set to 1

The concentrations of the bile lipids are summarized in Fig. 4. The data indicated that the mice that were fed an LD may have a great possibility of increasing bile indicators, such as TC, PL and CSI, all of which had significantly higher concentrations in the 8 + LD group and the 4 + LD group in comparison with the 0 + LD group. In the LD + UDCA group, the levels of TC, PL and CSI in the bile were significantly reduced when compared with the model group. All these results demonstrated that UDCA may play a key role in improving the CSI and bile lipids.

### The expression of lipid-related genes in the liver

**Fig. 5:**
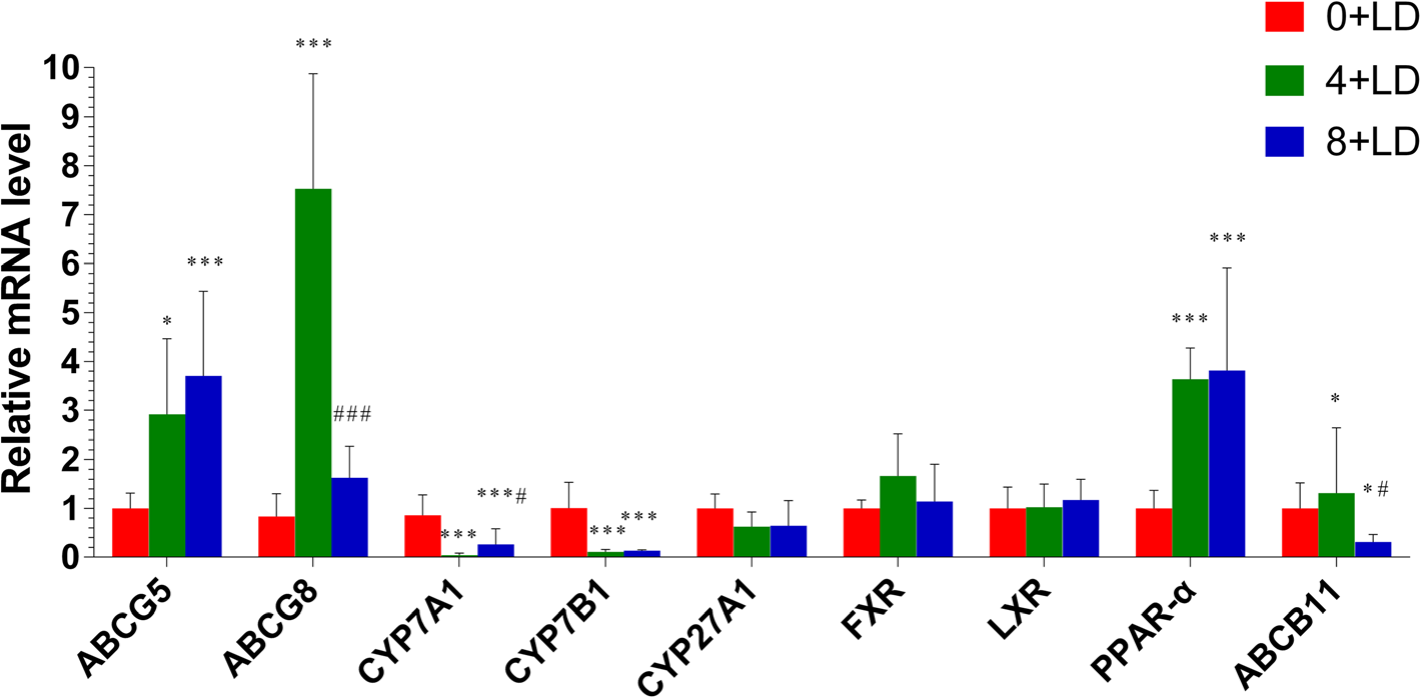
Relative mRNA expression levels of lipid-related genes in the 0 + LD group, 4 + LD group and 8 + LD group assessed using RT-PCR. *, compared to the 0 + LD group; #, compared to the 4 + LD group; *, #, *P* < 0.05; **, ##, *P* < 0.01; ***, ###, *P* < 0.001

Mice in the 4 + LD and 8 + LD groups showed higher expression levels of ABCG5 (*P* = 0.002) and PPAR-α (*P* < 0.001) with respect to the 0 + LD group, as shown in the results in Fig. 5. The expression levels of CYP7A1 (*P* < 0.001) and CYP7B1 (*P* < 0.001) showed significant reductions in the 4 + LD group and elevations in the 8 + LD group, conversely. ABCG8 (*P* < 0.001) was upregulated in the 4 + LD group and downregulated in the 8 + LD group. The expression levels of ABCB11 mRNA among the 0 + LD, 4 + LD, and 8 + LD groups were significantly different (*P* = 0.02).

**Fig. 6:**
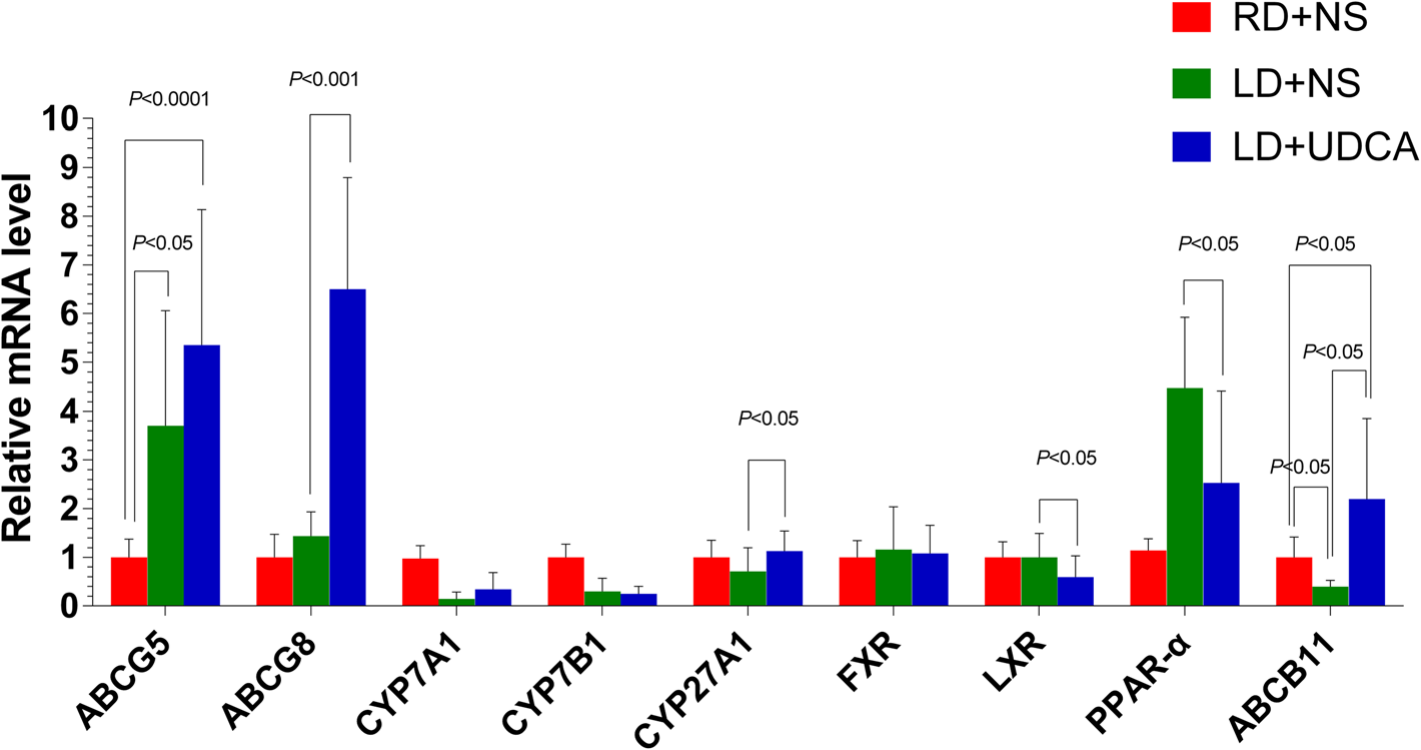
Relative mRNA expression levels of lipid-related genes in the RD + NS group, LD + NS group and LD + UDCA group assessed using RT-PCR

The results showed that there was a higher level of ABCG8 (*P* < 0.001) and CYP27A1 (*P* = 0.033), as well as a lower level of LXR (*P* = 0.026) and PPAR-α (*P* = 0.015) in the LD + UDCA group compared to the LD + NS group. The expression of ABCB11 mRNA among the LD + NS, RD + NS and LD + UDCA groups were also statistically significant (*P* = 0.008). However, there was no significant difference in the expression of ABCG5, CYP7A1, CYP7B1, and FXR between the LD + NS group and the LD + UDCA group (Fig. 6).

**Fig. 7:**
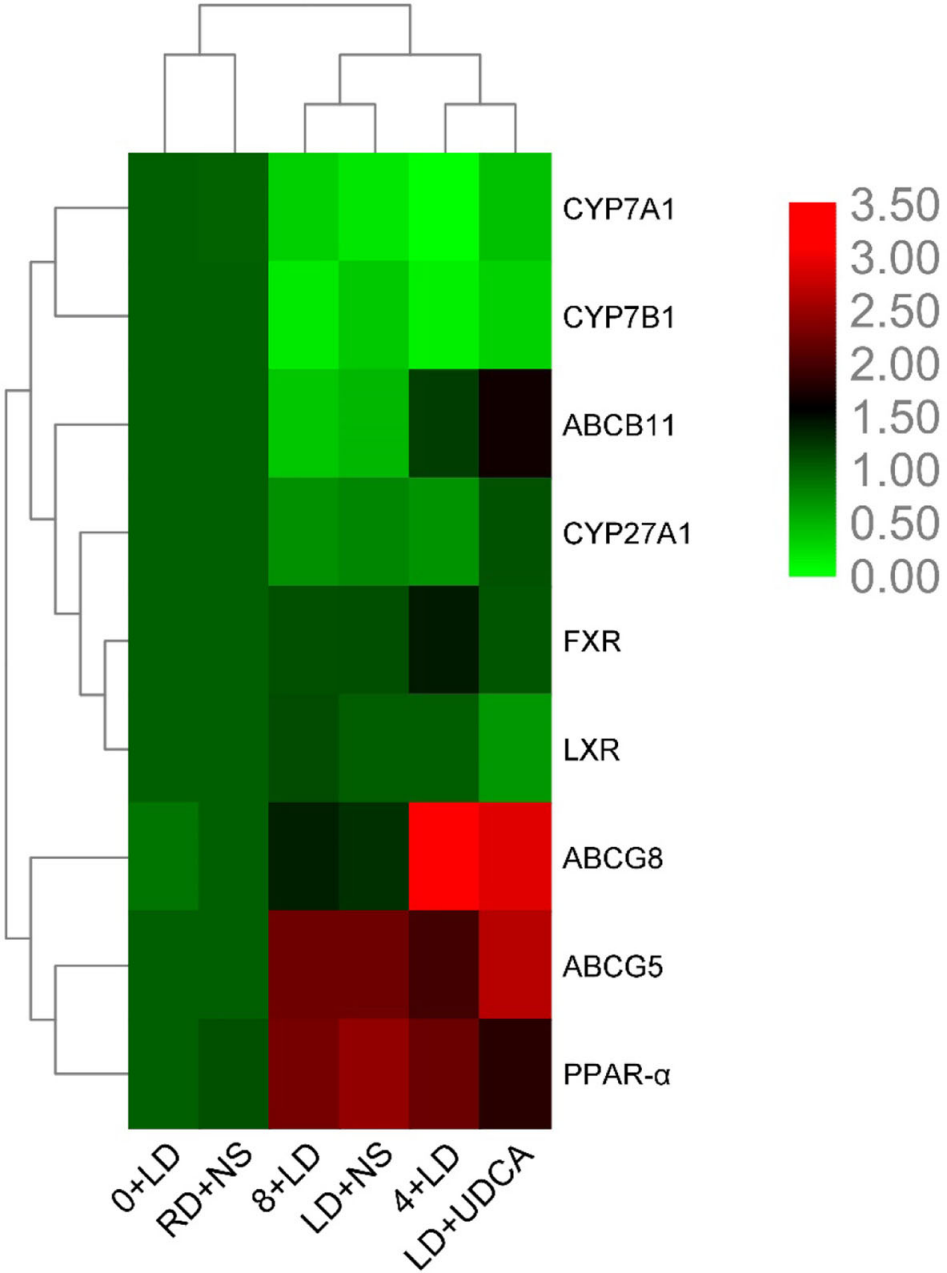
Heatmap representation of mRNA expression levels

The hierarchical cluster analysis and heatmap representation of mRNA expression levels in ABCG5/8, ABCB11, CYP7A1, CYP7B1, CYP27A1, FXR, LXR, and PPAR-α (Fig. 7).

**Fig. 8:**
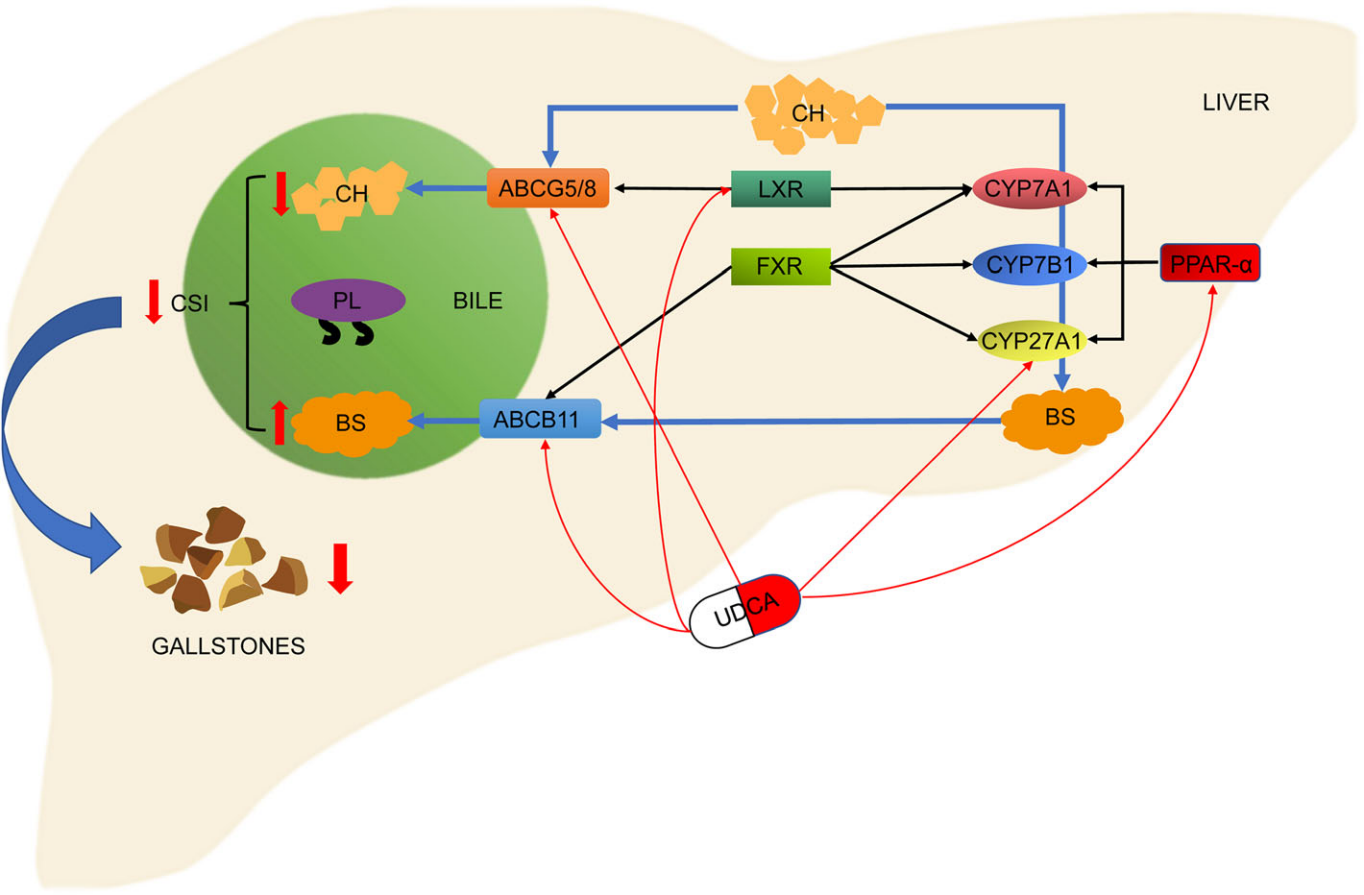
The figure shows the schematic diagram of cholesterol (CH) and bile salt (BS) metabolism regulated by the genes involved in this study, as well as the genes that UDCA might affect

The UDCA-dependent gene expression relationship is shown in Fig. 8.

### The correlation analysis between gene expression and bile lipids

A Spearman correlation coefficient analysis was performed to correlate bile lipid concentrations with gene expression levels. The results showed that ABCG5 and ABCG8 positively correlated with TC, PL and TBA in bile, CYP7A1 and CYP7B1 negatively correlated with TC, PL and TBA, and PPAR-α positively correlated with TC and PL, all of which were statistically significant differences (Table 2).

**Table 2:** Correlation analysis between gene expression and bile lipids

Bile lipid	ABCG5		ABCG8		CYP7A1		CYP7B1		CYP27A1		FXR		LXR		PPAR-α	
	r	*P*	r	*P*	r	*P*	r	*P*	r	*P*	r	*P*	r	*P*	r	*P*
TC	0.783	0.000	0.477	0.014	−0.618	0.001	−0.633	0.001	−0.134	0.515	−0.060	0.772	0.132	0.520	0.696	0.000
PL	0.617	0.001	0.467	0.016	−0.628	0.001	−0.618	0.001	−0.259	0.202	0.031	0.886	0.204	0.318	0.726	0.000
TBA	0.613	0.001	0.674	0.000	−0.534	0.005	−0.453	0.020	0.077	0.710	−0.142	0.490	−0.107	0.603	0.253	0.212

## Discussion

In this study, gallstone-susceptible C57BL/J mice successfully expressed a 100% gallstone prevalence after feeding on an LD for 8 weeks. The results of this study are consistent with those of Liu [23]. Due to its abundance of fat and cholesterol, a lithogenic diet induced cholesterol gallstones in the mice by progressively influencing the imbalance of lipid metabolism with higher TC and PL, thus giving rise to a higher bile CSI and cholesterol crystallization until gallstone formation and acceleration of the deterioration of the liver into fatty liver occurred. The gallstone prevalence and liver deterioration were gradually increased with the extension of feeding time. Of note, mice that had been administered UDCA showed a lower gallstone prevalence of 30%. These results reinforce the findings from previous studies that UDCA may exhibit a tremendously potent activity in increasing the bile acid pool size and thus reduce the cholesterol saturation index and cholesterol gallstone formation.

ABCG5/8 gene products, predominantly localized on the canalicular membrane of hepatocytes, are heterodimerized to function as a sterol transporter to facilitate cholesterol secretion into the bile canaliculus. The ABCG5/8 genes are transcriptionally regulated under the activation of LXR, and the overexpression of the ABCG5/8 genes is attributed to GSD [24–26]. Biliary cholesterol secretion is primarily maintained by ABCG5/8, while recent investigation insists that almost 30% of the cholesterol output is transferred via an ABCG5/8-independent pathway [27]. Lithogenic food could induce the excess secretion of cholesterol and oxysterol congeners, thus adversely affecting the expression of ABCG5/8.

The expression of ABCG5 mRNA in the 4 + LD and 8 + LD groups showed a significantly higher tendency with respect to the 0 + LD group. Although the expression of LXR increased gradually with the extension of LD feeding time, the difference was not significant. For ABCG8, there was a significantly increased level in the 4 + LD group and a decrease in the 8 + LD group. Conversely, UDCA may have a potential ability to enhance the transcription of ABCG8 and to decrease LXR, compared to the model group. Bile acids are thought to regulate their own homeostasis via their sensor, the FXR, which could be directly activated by UDCA [28]. LXR are “cholesterol sensors” which, in response to excess cholesterol, stimulate its transport to the liver and biliary excretion [17]. The possible mechanism for this is that when UDCA enters the bile, it changes the bile salt concentration and increases cholesterol solubility. On one hand, UDCA can dissolve cholesterol stones/crystals in bile, and on the other hand, it can also stimulate ABCG8 to transport cholesterol forward into the bile. Therefore, the expression of ABCG8 is increased. LXR, as a cholesterol receptor, was passively downregulated to maintain cholesterol homeostasis when cholesterol transport increased. In addition, studies have shown that the ABCG5/8-independent pathway plays a critical role in regulating hepatic cholesterol secretion and in determining the susceptibility to cholesterol gallstones, working independently of the ABCG5/8 pathway, and that the hepatic LXR does not have an effect on the ABCG5/8-independent pathway for regulating biliary cholesterol secretion [29]. This is different from the classical mechanism of the LXR-ABCG5/8 pathway in regulating cholesterol metabolism. Because the concentration of bile salt is limited, when the concentration of bile salt reaches a constant level, it can affect the solubility of cholesterol, leading to cholesterol crystallization/stone formation. It may also be that UDCA is not effective for every patient or that stones recur after a period of time. Of course, many studies are needed to verify this in a follow-up study.

It is known that there are two pathways for the elimination of redundant cholesterol, where one is via the LXR-ABCG5/8 pathway to bile and the other is via bile acid synthesis to convert cholesterol into bile acid. One possible reason for the results obtained is that in response to persistent hypersecretion of cholesterol or oxycholesterol, the feed-forward loop may become active and the LXR mRNA may be motivated to decrease the cholesterol level in hepatocytes [30]. An increase in cholesterol can lead to an increase in oxysterols, which activates LXR to induce CYP7A1 and ABCG5/8 expression. The activation of LXR significantly influences ABCG5/8, thus increasing the transcription of ABCG5/8. The possible reason is that ABCG8 may contribute to the peak of the lithogenic effect after 4 weeks of lithogenic food. When the mice were fed with LD for 4 weeks, the expression of ABCG8 increased significantly, which was associated with the activation of this classical pathway. However, it is important to mention that LXR only induces CYP7A1 in rats and mice and not in hamsters, rabbits or humans [31].

It is known that excess cholesterol may participate in bile acid synthesis, which is coordinated by CYP7A1 and CYP27A1 [32]. These are two critical enzymes in the classic and alternative pathways to regulate cholesterol trafficking into CDCA or CA, which are concomitant with their downstream target genes to sustain cholesterol homeostasis. ABCB11 is a bile salt export pump that facilitates the efflux of bile acid to the canalicular membrane, thereby performing enterohepatic circulation of bile acid. Additionally, it was widely believed that the upregulation of LXR may exert a beneficial effect on the elevation of CYP7A1 expression, while FXR as a bile acid receptor may suppress CYP7A1 [33, 34]; however, in this experiment, no significant difference was observed in the FXR mRNA. It is thus controversial whether or not PPAR-α may downregulate CYP7A1 and CYP27A1 mRNA in fatty acid catabolism [35–37]. In the present study, the results suggested that the upregulation of PPAR-α may be implicated in the downregulation of CYP7A1. This is consistent with Xie’s research results [38]. In the LD-fed L-Fabp−/− mice, in the absence of L-Fabp (whose expression is generally regulated in a proximal to distal gradient), alterations in intestinal BA flux induce FXR expression, which results in transcriptional induction of downstream targets, including fibroblast growth factor (FGF) 15 [39]. Increased intestinal FGF15 expression would in turn be anticipated to suppress hepatic CYP7A1 expression [40, 41]. Thereafter, there was a remarkable finding that CYP7A1 and CYP7B1 mRNA showed downregulation at 4 weeks and, conversely, upregulation at 8 weeks, while PPAR-α may show a sustainable increase from 0 to 8 weeks. A different discrepancy in CYP7A1 may be attributed to the common control of LXR, FXR and PPAR-α, while CYP27A1 may be affected by the higher expression of PPAR-α. A prolonged lithogenic diet may produce serious injury to hepatocytes or the canalicular membrane, thus leading to the decrease in the CYP7A1, CYP7B1 or CYP27A1 translocators. Recently, studies have shown that UDCA may be an FXR antagonist [42, 43]. In liver, the majority of UDCA is conjugated with glycine and taurine to produce glycoursodeoxycholic acid (GUDCA) and tauroursodeoxycholic acid (TUDCA), which are transported into the gut and then reabsorbed into the ileum epithelial cells. This study revealed that GUDCA and TUDCA were bona fide FXR antagonists. Oral administration of GUDCA suppressed FXR signaling in the gut but did not affect FXR signaling in the liver. Because UDCA was found not to be a direct FXR antagonist, the metabolic benefits of UDCA may be due in part to GUDCA [44].

ABCB11 mRNA expression showed a trend of an increase first, followed by a decrease, among the 0 + LD, 4 + LD, and 8 + LD groups. This may be related to the influence of other factors on the expression of ABCB11, such as steroid receptor coactivator-2 (SRC-2), liver kinase B1 (LKB1), liver receptor homolog 1 (Lrh1), NF-E2-related factor-2 (Nrf2), and others [45–47]. Moreover, the expression of ABCB11 mRNA in the LD + UDCA group was significantly higher than that in the RD + NS and LD + NS groups. This indicated that under UDCA intervention, the expression of ABCB11 mRNA was upregulated, which was consistent with the results of Hu et al. The ABCB11 gene encodes a protein called the bile salt export pump, which transports bile salts from the hepatocyte into bile. The overexpression of ABCB11 significantly promoted biliary bile salt secretion and increased the circulating bile salt pool size and bile salt-dependent bile flow rate [48]. After that, cholesterol solubility can be increased, thus lowering the CSI and reducing cholesterol stone formation. The increased expression of ABCB11 could increase the rate of transport of bile salts within the hepatocyte, thereby decreasing their steady-state intracellular concentration and thus leading to enhanced signaling [49]. As a natural ligand of liver FXR, bile acid can play a role as a signaling molecule regulating liver metabolism [50]. Further studies are needed to confirm the possibility that lipid changes in bile may affect the expression of some genes.

To date, it has been discovered that the CYP27A1, LXR, PPAR-α, ABCG8 and ABCB11 genes involved in lipid metabolism predispose an individual to gallstone formation [51, 52]. However, their interactions with UDCA have not been fully ascertained yet.

### Strength and study limitation

This study only examined gene expression in liver tissue and did not detect the same genes or undetected target genes in sites such as the gallbladder and intestines. This study was restricted to in vitro experiments, and the protein levels of the lipid-related genes should be further determined to verify the conclusions.

In the future, more basic experiments are needed to study the specific physiological mechanism of UDCA treatment of cholesterol stones. The protein levels of these genes need to be tested, and it needs to be confirmed whether these results in vitro have potential value for research on the mechanism, prevention and treatment of human cholelithiasis. Future research should ultimately be concentrated on the definitive molecular and pharmacokinetic mechanism by which UDCA may affect certain lipid-related genes to assess the therapeutic feasibility of gene agonists that may be applied to GSD.

## Conclusions

With regard to the mouse groups on an LD for 0, 4, and 8 weeks, it should be emphasized that these alterations in the lipid-related genes may accelerate cholesterol accumulation and bile supersaturation while modulating bile lipids and the CSI to exert significant effects on gallstone formation. UDCA may achieve a cholagogic effect by decreasing the levels of TC, PL and CSI to normalize lipid composition. Thus, increasing the expression levels of ABCG8, ABCB11, CYP7A1 and CYP27A1 or decreasing the levels of LXR and PPAR-α can stimulate bile acid secretion and efficiently facilitate gallstone dissolution to reverse damage to the hepatocytes. UDCA may play a role in regulating the expression levels of some lipid-related genes directly or indirectly to ameliorate the development of gallstones. Therefore, UDCA can be used as the drug of choice for cholesterol gallstones if patients are unwilling to undergo surgery.

## Data Availability

The datasets used and/or analysed during the current study are available from the corresponding author on reasonable request.

## Abbreviations

UDCA: Ursodeoxycholic acid
LD: Lithogenic diet
CSI: Cholesterol saturation index
RT-PCR: Real-time polymerase chain reaction
GSD: Gallstone disease
FXR: Farnesoid X receptor
LXR: Liver X receptor
PPAR-α: Peroxisome proliferator activated receptor alpha
CYP7A1: Cholesterol 7-α hydroxylase
CYP27A1: Sterol 27-α hydroxylase
ABCB11: Adenosine triphosphate-binding cassette subfamily B member 11
CDCA: Chenodeoxycholic acid
CA: Cholic acid
ABCG5: Adenosine triphosphate-binding cassette subfamily G member 5
ABCG8: Adenosine triphosphate-binding cassette subfamily G member 8
ABCB4: Adenosine triphosphate-binding cassette subfamily B member 4
PL: Phospholipids
TBA: Total bile acid
NS: Normal saline
RD: Regular rodent diet
HE: Hematoxylin eosin
TC: Total cholesterol
GAPDH: Glyceraldehydes-3-phosphate dehydrogenase
LSD: Least significant difference
SNK: Student-newman-keuls
CYP7B1: Oxysterol 7-α hydroxylase
FGF15: Fibroblast growth factor 15
GUDCA: Glycoursodeoxycholic acid
TUDCA: Tauroursodeoxycholic acid
SRC-2: Steroid receptor coactivator-2
LKB1: Liver kinase B1)
Lrh1: LIVER receptor homolog 1
Nrf2: NF-E2-related factor-2
CH: Cholesterol
BS: Bile salt
